# Rapid Breathing

**Published:** 1881-09

**Authors:** Addinell Hewson


### Selections.

### RAPID BREATHING.

By Addinell Hewson, Senior, A. M., M. D.,

My pursuit of Bonwill's method of producing "*insensibility to pain*," ever since he first directed my attention to the subject, some ten years ago, has afforded me a collection of very curious as well as interesting experiences, from which I now propose to communicate those of most practical value to the profession.

The process itself consists essentially of rapid—excessively rapid—respirations, kept up continuously for some minutes, say from three to five or seven, to induce the desired state, while the patient's face is covered by a handkerchief or towel hanging from the forehead, so that there may be no diversion of attention by what may be in the room or going on there. This breathing must be kept up at as high a rate of speed as possible; this is often easiest accomplished by directing the patient to blow out his breath as fast as he can. The effect of this is soon manifest by the patient's manner, his seeming weary, and his needing urging on more constantly. A pinch in the flesh now, say on the hand, arm, neck, or elsewhere most accessible, will provoke a sigh, a lament, or a positive exclamation, either of which will have its definite signification, easily recognized; the last showing perfect *sensibility to pain;* the first, the loss of it; the second, only its impairment. These different states may also be determined by directly asking the patient, not "whether he feels it" (the pinch), for he never loses his *sense of touch*, and will, therefore, always say "yes" to that question, but "whether it hurts him," and if he says "yes," he is not yet "insensible to pain;" if, on the contrary, he says "no," then the state of "*insensibility to pain*" has been brought about, and needs only the continuance of the "rapid breathing" to keep it on the patient as long as you need it for the purpose of your resorting to it.

In communications which I have before made to the profession on this subject I have occasion to refer to the advantages of this mode of provoking "insensibility to pain," in certain instances, over those of ether, or chloroform—where true anæsthesia is induced but not necessary. My communication now will also be relative to the same, and I will proceed, without further preliminary remark, to make that for to-day in a sketch of a single case, that of Mr. ——, aged ——, over — feet high, who had been a fine looking and a vigorous man in his day. I found him on one occasion, in my front office, waiting his turn, a picture of decrepitude and woe, his tall figure doubled up in a heap on a sofa—his head, with its thin covering of perfectly white hair, projecting forward, with agony marked in his countenance, and his hands shaking violently.

I had seen him often in the past ten years—when I was attending some of his family at his home, and had there acquired the impression that he suffered with senile cystic trouble. This time he was before me, evidently the victim of severe cramp, and as soon as I could get him into my back office—for I had to help him there—he walked around it all doubled up, wringing his hands, and saying he was suffering with too great pain in his bladder to talk to me.

I immediately made him sit down on a chair, threw a napkin over his face, and directed him to blow out his breath as fast as he could. This he did without any hesitation, and—by my watch—in four minutes' time he said, "Oh! (with a deep inspiration) it is gone now, let me go to your water-closet." There he emptied his bladder promptly, and returning to my room expressing himself perfectly delighted, said, "My God! what a blessing that rapid breathing is."

I saw him frequently after this, and he always gave vent to the same sentiment, saying, also, that he never failed to resort to this breathing as soon as he felt cramps coming on, and so succeeded in checking the pain.

"Rapid Breathing" has been shown, also, by the discoverer of its effects on pain, to leave the will and consciousness untrammeled; so that one under it knows what is going on around, and can be induced to co-operate in the matter of changing position, and other muscular exertions that may be desirable in many instances, and the want of which co-operation is often seriously felt by the surgeon with his patient under the influence of ether or chloroform. Thus, in the use of the catheter—male, or female—in the examinations of the rectum, or vagina, either *per digitum*, or *per speculum*, and even in actual cutting operations of those parts, as for stricture, fistula, or the like—in the latter, the knowledge of the fact that a vast majority of fatal results from chloroform have occurred from its use in them—gives a surgeon extreme satisfaction from security against such with the "Rapid Breathing."

So, in labor itself, one has not only the advantages cited above as belonging to rapid breathing, but is secured against the after-effects even of ether in it, and which have been shown to increase the mortality of normal labors. Here I will introduce my "clinical fact" for this communication.

Mrs. ——, aged thirty-four, had been, in girl and early womanhood, a wild and independent creature in the country, riding bareback and the like. Her menses came on at about thirteen, with great suffering, and recurred regularly in the same manner, but grew worse until the reflex irritations and hysteria were attended with convulsion and even extreme opisthotonos during the immediate antecedents of the flux—then they came on more or less constantly, until at the end of about six years from the beginning of this extreme state, when they were positively constant. I then saw her for the first time, promptly recognized the source of her trouble in the womb, and succeeded, after some persuation, in getting her and her parents' consent to my examining her there. An elongated neck and contracted os, with displacement, great hyperæsthesia, and the examination provoking one of her most violent spasms of the erector spinæ mass, confirmed me in my notion, and I was not very long in bringing about a cure of those troubles, and that, too, chiefly by local treatment. This cure was a permanent one, and three years after it was effected the lady married, promptly became pregnant, and was delivered by me, exactly nine calendar months and twenty-one days from the date of her marriage, of a fine, healthy, ten-pound boy. This delivery was by means of ether and the forceps, just three hours after the first pain was felt. I began the use of the ether as soon as I got to the house, which was but half an hour after the labor began. The patient, her husband and her mother, all unacquainted with ether, opposed very much my resorting to it so early. They were afraid of some bad effects; none, however, followed, save the ordinary sequences of headache, nausea, etc., extending through the next day. Great care was taken in her getting up. Her milk came without any trouble, and she nursed her baby for over eight months, when his failing condition led to the recognition of the pregnant state in the mother. Conception had taken place without any preceding menstrual show. About the time of its occurrence, as was determined afterward, the mother showed some disposition to nervousness, which she had never done before during the whole time of her lactation. She went through this second pregnancy to its full term without any nervous spells or any kind of annoyance whatever, and when I was called to her, on the setting in of the labor, I was asked to try some other means than ether, so that she might escape its after annoyances as experienced before.

Her folks speaking of having heard that the use of chloroform was not followed by such, I promised to use something better than even chloroform in that respect. I at once determined, by a digital exploration of the os, that the labor had actively begun, desired my patient to remain on her left side, and, covering her face with a towel, directed her to breathe, or blow out, as rapidly as she could. Then, turning to an attendant, I asked for a pitcher of warm water, and in it I placed a pair of long forceps. This act on my part was noticed by the patient, and yet, notwithstanding this circumstance, in eight minutes' time from my doing this act of putting the forceps in the pitcher, as was shown by a large clock on the mantel, I had my patient not only insensible to pain, and her os fully dilatable, but the instrument applied in the upper strait, the baby born, and the afterbirth away, so that there was then no longer any need for the rapid breathing, and I left the room, forbidding all talking or explanation about what had taken place. At my next visit she was enthusiastic about *rapid breathing*, but was not prepared to believe that she had been delivered by instrument, and, that too, in so short a time.

She was even more fortunate with this child than with the first. She got him through two summers without having to wean him, and did not have her next (her third) conception until two years and three months after this second one's birth.

This was also carried to full term, and when the labor for it set in, I was, as usual, summoned early, and went armed with my instruments. When I got into her chamber I found the patient hard at work, as she said, "blowing away all suffering from the pains." I said, "go on!" and made my digital examination of the os at once, before the paroxysm then on was over, and so determined the head with vertex to the right, merely held up by the anterior lip of the os. When this paroxysm ceased, my patient exclaimed "Oh, what a luxury this breathing is." I said "you must not talk now, but go on with the breathing," and as soon as she became insensible to pain again I found the head readily disengaged and expelled from the uterus. I then let the labor go, without further interference, simply urging the patient constantly on with the rapid breathing; and in twenty minutes, by the same clock, it was all over, and we had another well-developed boy.

As soon as the mother was told to take a good deep breath, that it was all over, and had done so, she exclaimed, "My! how much more sensible it is for you always to use the instruments at once without any waiting!" I said, "what, I did not use the instruments this time!" "Oh yes!" she said, "I felt them just as plain this time as before; however, they never gave me pain, this or any other time."—*The College and Clinical Record.*

